# Knowledge landscapes and emerging trends of cardiorenal syndrome type 4: a bibliometrics and visual analysis from 2004 to 2022

**DOI:** 10.1007/s11255-023-03680-4

**Published:** 2023-07-09

**Authors:** Han Li, Tongtong Liu, Liping Yang, Fang Ma, Yuyang Wang, Yongli Zhan, Huimin Mao

**Affiliations:** 1 Guang’anmen Hospital, China Academy of Chinese Medical Sciences, Beijing, 100053 China; 2 Beijing University of Chinese Medicine, Beijing, 100029 China

**Keywords:** Bibliometric analysis, Cardiorenal syndrome type 4, CiteSpace, VOSviewer, Bibliometrix

## Abstract

**Purpose:**

To evaluate the key topics and emerging trends in the field of cardiorenal syndrome type 4 (CRS-4) by bibliometrics and visual analysis.

**Methods:**

Citespace, VOSviewer, and Bibliometrix package were used to analyze the collected data from the Web of Science Core Collection, including publication trends, leading countries, active authors and institutions, co-cited references, journals, and keyword analysis.

**Results:**

Finally, 2267 articles were obtained. From 2004 to 2022, the number of publications was increasing year by year. A total of 735 authors from 543 institutions in 94 countries/regions participated in the publication of CRS-4 field, which were mostly from North America and Europe. Most of the co-cited references were reviews or guidelines from kidney/heart specialist journals or top journals. The journals concerning nephrology had a higher academic influence in this field. Oxidative stress and inflammation remained hot topics in CRS-4 research, as well as uremic toxins. Fibroblast growth factor 23 and klotho were emerging trends in recent years. Sodium glucose cotransporter 2 (SGLT2) inhibitors were the latest frontier hot spots. Future research advances may pay more attention to the prevention and prognosis assessment of CRS-4.

**Conclusion:**

Our study provides some key information for scholars to determine the direction of future research.

## Introduction

Chronic kidney disease (CKD) has become a worldwide health problem with an increasing prevalence. According to the Global Burden of Disease study, there were 697.5 million CKD cases worldwide in 2017, with a prevalence of approximately 9.1% [1]. Patients with CKD exhibit a great risk for cardiovascular diseases (CVD), and cardiovascular mortality increases with deteriorating kidney function [2]. At the same time, patients with CVD also have a higher prevalence of concurrent CKD. About 40–50% of patients with heart failure (HF) are accompanied with renal dysfunction [3]. The coexistence of CKD and CVD has significantly reduced the quality of life of patients and increased the medical and economic burden globally, which has attracted extensive attention from scholars.

Scholars' concerns about the interaction between kidney and heart first emerged in 1836 [4]. Since then, heated discussions have been launched in this field. In 2010, Ronco et al. defined cardiorenal syndrome (CRS) as a clinical syndrome in which acute or chronic failure of one organ (heart or kidney) leads to acute or chronic failure of another organ (kidney or heart) in the Acute Dialysis Quality Initiative (ADQI) consensus group. Cardiorenal syndrome type 4 (CRS-4) we focus on refers to cardiac damage occurring secondary to CKD, including coronary heart disease (CHD) (such as left ventricular remodeling and dysfunction, diastolic dysfunction, abnormalities in cardiac function, etc.), HF and acute coronary syndrome (ACS) [5].

Bibliometrics is a subject that applies mathematical and statistical methods to analyse quantitatively of literature information [6]. CiteSpace [7], VOSviewer [8], and Bibliometrix [9] are three commonly-used scientific bibliometric software. The analysis and prediction of the development trend in a field have gradually become an important means for researchers to focus on research directions through the co-occurrence and co-citation analysis of the existing literature in that field [10].

In the present study, we aim to evaluate the key topics and emerging trends in the CRS-4 field in order to further guide clinical practice.

## Materials and methods

### Search strategy and data collection

Search for literature on CRS-4 in the Web of Science Core Collection (WoSCC) from inception to November 22, 2022. We limited the type of literature to “papers” or “review papers”, and limited the language to “English”. Based on the concept of CRS-4, we determine the search formula as follows:
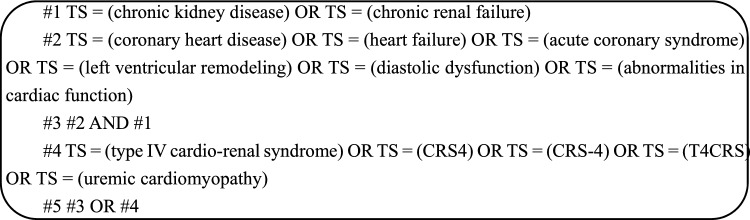


In this study, we defined CKD events as CKD patients or animal models, CKD-related clinical manifestations and complications. At the same time, we defined cardiovascular events as heart injury, disease, and/or dysfunction. Based on this, the inclusion and exclusion criteria were established as follows:

1. Inclusion Criteria: Reviews involving cardiovascular events occurring in CKD patients or CRS-4 or uremic cardiomyopathy; experimental studies with CRS-4 or uremic cardiomyopathy as subjects; experimental reports concerning cardiovascular events occurring in CKD patients.
2. Excluded Criteria: Studies involved both CKD events and cardiovascular events, but didn't focus on the relationship between the two; studies only covered a wide range of CRS, but didn't mention the specific type of CRS-4; research without the full text or complete author and year information; repeated published literature.

Due on November 22, 2022, we retrieved 15,836 articles. We excluded 13,566 articles, deleted 3 duplicate articles, and finally included 2267 articles for quantitative analysis. The time span of the included literature was 2004–2022.

### Data analysis and software

Citespace (version 6.1.R3), VOSviewer (version 1.6.18) and Bibliometrix 4.2.2 package (https://www.bibliometrix.org) were used to analyze the collected data, including publication trends, leading countries, active authors and institutions, co-cited references, journals and keyword analysis.

Citespace was used to map the distribution network, while clustering, timeline view, and burst analysis were performed. In the spectrum, the frequency of occurrence was measured by the node size, and the thickness of the link between the two nodes was proportional to the frequency of co-occurrence. Blue rings indicated the earlier years and yellow rings indicated the closer years. Centrality ≥ 0.1 represented the importance of node, which were highlighted with purple circles.

VOSviewer was also used to analyze knowledge areas of CRS-4, including extracting and visualizing the national start time and keywords. Unlike Citespace, the same node color indicates close cooperation, not time.

The Bibliometrix package was used to analyze the number of publications, delineate core journals, and construct the topic map and evolution of keywords. The time cutting points were set to 2013 and 2018 (recent 10 years and 5 years).

## Results

### Publication trends

**Fig. 1:**
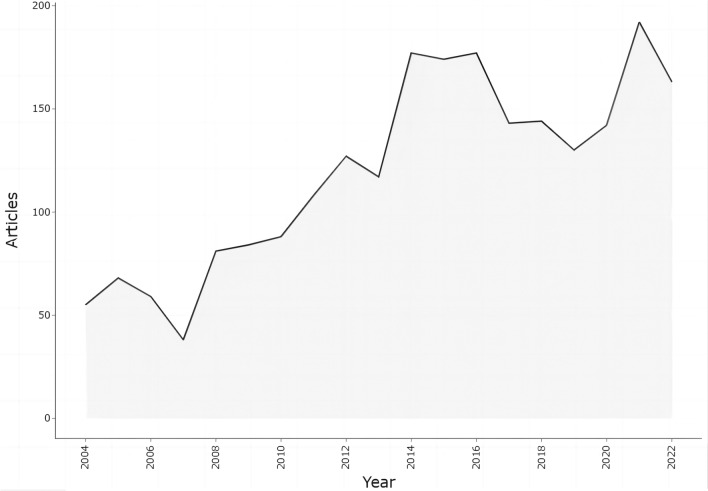
Annual publication volume of CRS-4 (2004–2022)

From 2004 to 2022, the number of publications basically increased, with annual growth rate 6.22% (Fig. 1). After a small fluctuation in the early stage of the study, the number of papers published in this field increased dramatically, reaching 177 in 2014. Since then, scholars' enthusiasm for CRS-4 has reached a plateau.

### Leading country analysis

**Fig. 2:**
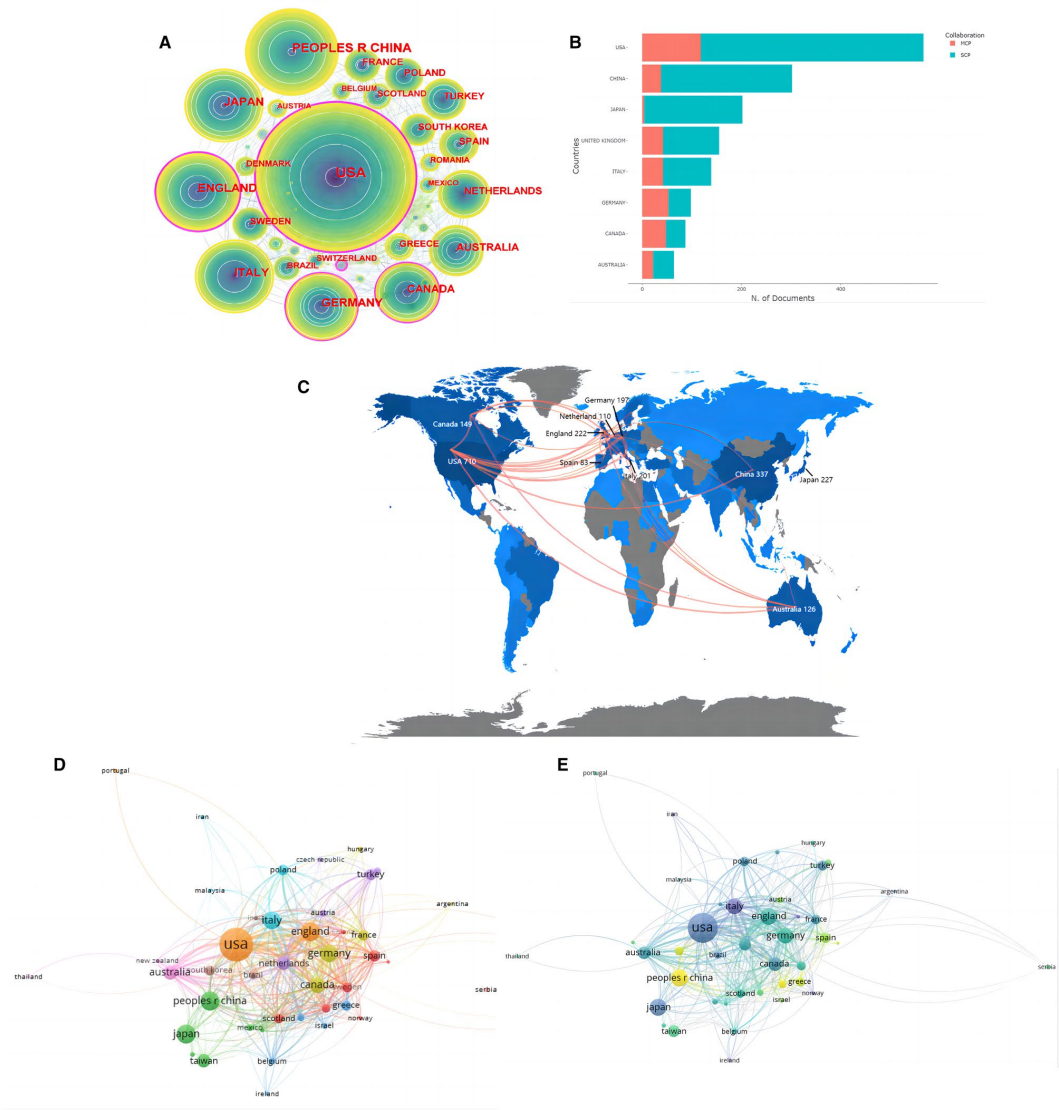
Leading country analysis. **A** National contributions. **B** Top 8 countries. **C** Global geographic distribution and leading countries. **D** Clustering of collaboration among countries. **E** Timeline view of collaboration among countries

Of all the 94 countries/regions, the largest contributor was the USA (710, 31.32% of all articles) (Fig. 2B). There were five countries with centrality ≥ 0.1, namely the USA (0.26), England (0.27), Germany (0.18), Canada (0.11), and Norway (0.27) (Fig. 2A). This was consistent with the close cooperation network between them (Fig. 2C). The USA dominated the largest cooperative network (Fig. 2D). China started late (Fig. 2E).

### Active authors and institutions analysis

**Fig. 3:**
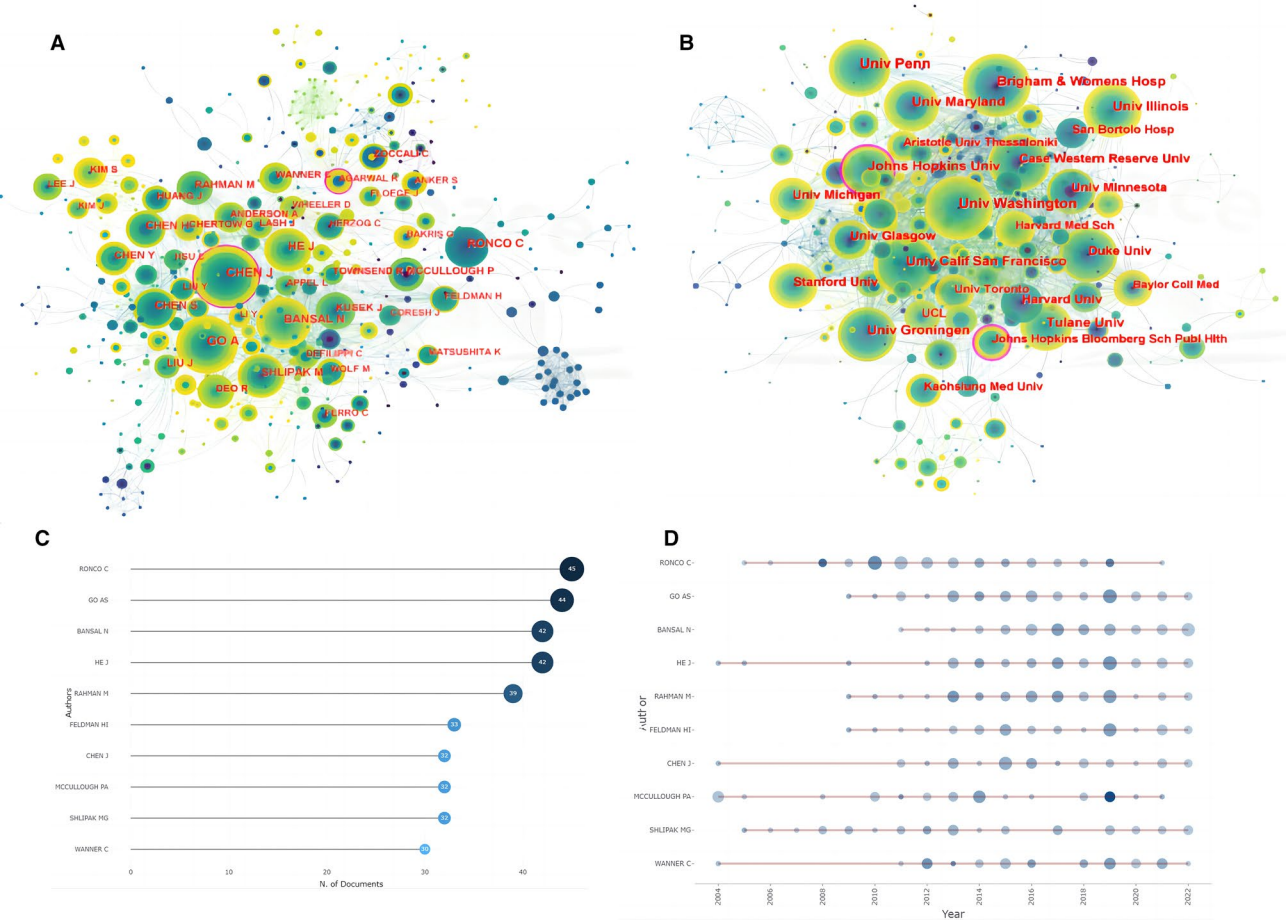
Active authors and institutions analysis. **A** Author collaboration network. **B** Institution collaboration network. **C** Top 10 authors. **D** Timeline distribution of the top 10 authors

A total of 736 authors from 543 institutions contributed to the study of CRS-4. The most influential authors were CHEN J and AGARWAL R (Fig. 3A). RONCO C contributed the most (45 articles, 2.0%). As a rising star, GO AS and BANSAL N had risen to the top three in terms of publication (Fig. 3C, D). Both institutions with centrality ≥ 0.1 were from Johns Hopkins University (Fig. 3B). The top 10 institutions contributed 23.16% of all outputs, of which 9 were from the USA (Table 1).

**Table 1:** Top 10 institutions in the field of CRS-4 (2004–2022)

NO	Institution	Year	Centrality	Counts	Percentage (N/2267) (%)
1	Univ Washington	2005	0.04	62	2.73
2	Univ Penn	2011	0.01	61	2.69
3	Univ Calif San Francisco	2009	0.04	54	2.38
4	Brigham & Womens Hosp	2004	0.07	45	1.99
5	Tulane Univ	2013	0	45	1.99
6	Univ Groningen	2010	0.08	44	1.94
7	Univ Maryland	2008	0.04	44	1.94
8	Johns Hopkins Univ	2008	0.13	43	1.90
9	Univ Illinois	2010	0.01	43	1.90
10	Duke Univ	2005	0.05	42	1.85
11	Stanford Univ	2011	0.08	42	1.85

### Co-cited reference network analysis

**Fig. 4:**
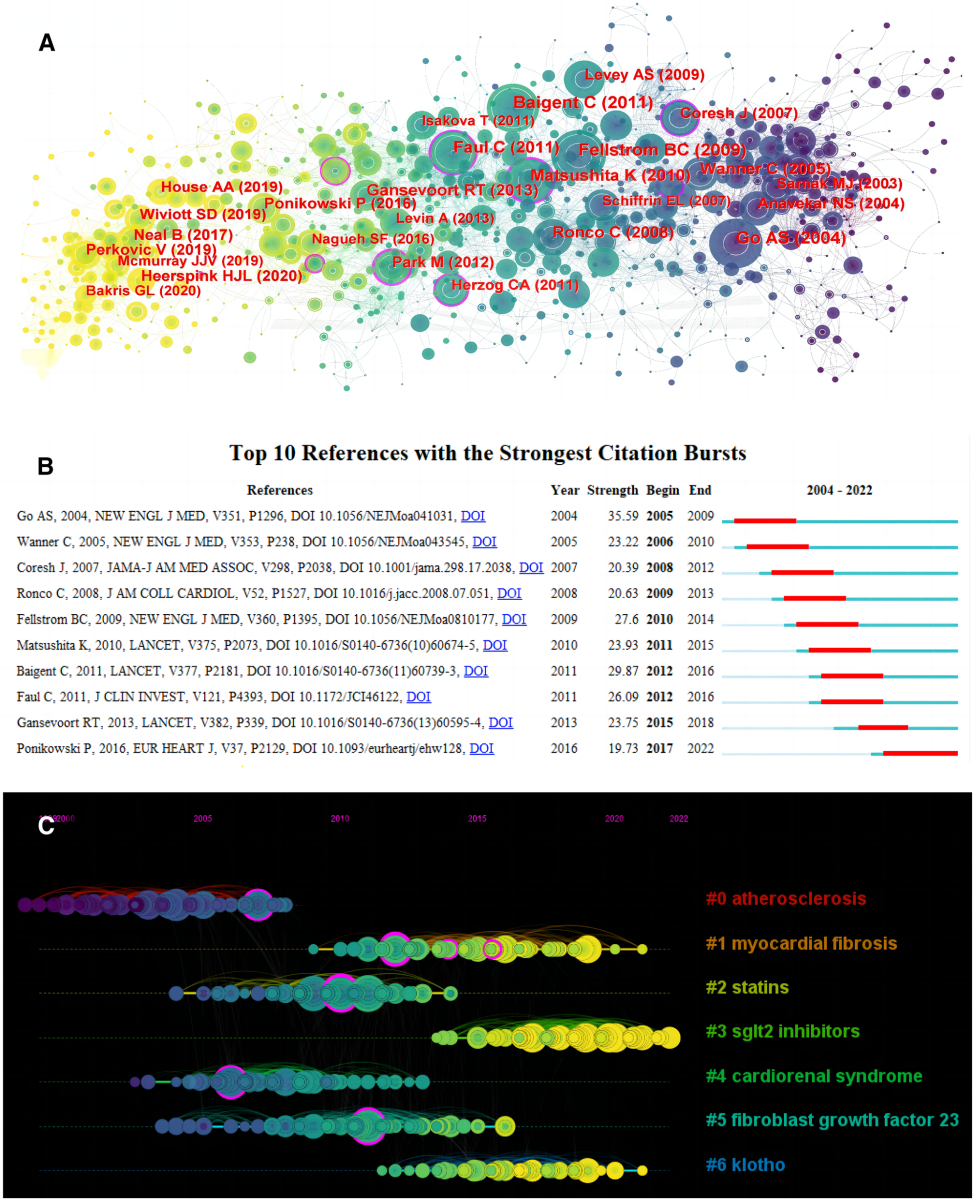
Co-cited reference network analysis. **A** Co-citation references network. **B** Burst analysis based on references. **C** Timeline plot of clustering based on references

The co-cited reference network contained 957 references (Fig. 4A). The most frequently cited reference was *Chronic kidney disease and the risks of death, cardiovascular events, and hospitalization* published in *The New England Journal of Medicine* [11]. The key node literature covered not only the risk assessment and epidemiology of CKD, but also the pathogenesis, diagnostic, prognosis, and treatment of CRS-4. In addition, we conducted a burst analysis of the references and obtained highly similar results with highly cited literature (Fig. 4B and Table 2).

**Table 2:** Top 10 references in the field of CRS-4 (2004–2022)

No.	References	Counts	Content
1	Go AS, 2004, NEW ENGL J MED, V351, P1296	74	Chronic kidney disease and the risks of death, cardiovascular events, and hospitalization [11]
2	Fellstrom BC, 2009, NEW ENGL J MED, V360, P1395	74	Rosuvastatin and cardiovascular events in patients undergoing hemodialysis [12]
3	Baigent C, 2011, LANCET, V377, P2181	72	The effects of lowering LDL cholesterol with simvastatin plus ezetimibe in patients with chronic kidney disease (Study of Heart and Renal Protection): a randomised placebo-controlled trial [13]
4	Faul C, 2011, J CLIN INVEST, V121, P4393	63	FGF23 induces left ventricular hypertrophy [14]
5	Matsushita K, 2010, LANCET, V375, P2073	58	Association of estimated glomerular filtration rate and albuminuria with all-cause and cardiovascular mortality in general population cohorts: a collaborative meta-analysis [15]
6	Wanner C, 2005, NEW ENGL J MED, V353, P238	48	Atorvastatin in patients with type 2 diabetes mellitus undergoing hemodialysis [16]
7	Gansevoort RT, 2013, LANCET, V382, P339	47	Chronic kidney disease and cardiovascular risk: epidemiology, mechanisms, and prevention [17]
8	Ronco C, 2008, J AM COLL CARDIOL, V52, P1527	47	Cardiorenal syndrome [18]
9	Coresh J, 2007, JAMA-J AM MED ASSOC, V298, P2038	46	Prevalence of chronic kidney disease in the United States [19]
10	Ponikowski P, 2016, EUR HEART J, V37, P2129	45	2016 ESC Guidelines for the diagnosis and treatment of acute and chronic heart failure: The Task Force for the diagnosis and treatment of acute and chronic heart failure of the European Society of Cardiology (ESC)Developed with the special contribution of the Heart Failure Association (HFA) of the ESC [20]

Next, among the 7 clusters obtained (Fig. 4C), the most widely studied clusters were #0 atherosclerosis and #1 myocardial fibrosis, pointing out the pathophysiological characteristics of CRS-4. Traditional pharmacotherapeutic studies had focused on #2 statins, which was supported by highly cited literature (Table 2). Cluster #3 sglt2 inhibitors was emerging therapeutic ideas that had been widely studied in recent years. Clusters #5 fibroblast growth factor 23 and #6 klotho marked the microscopic progress of research.

### Journal analysis

**Fig. 5:**
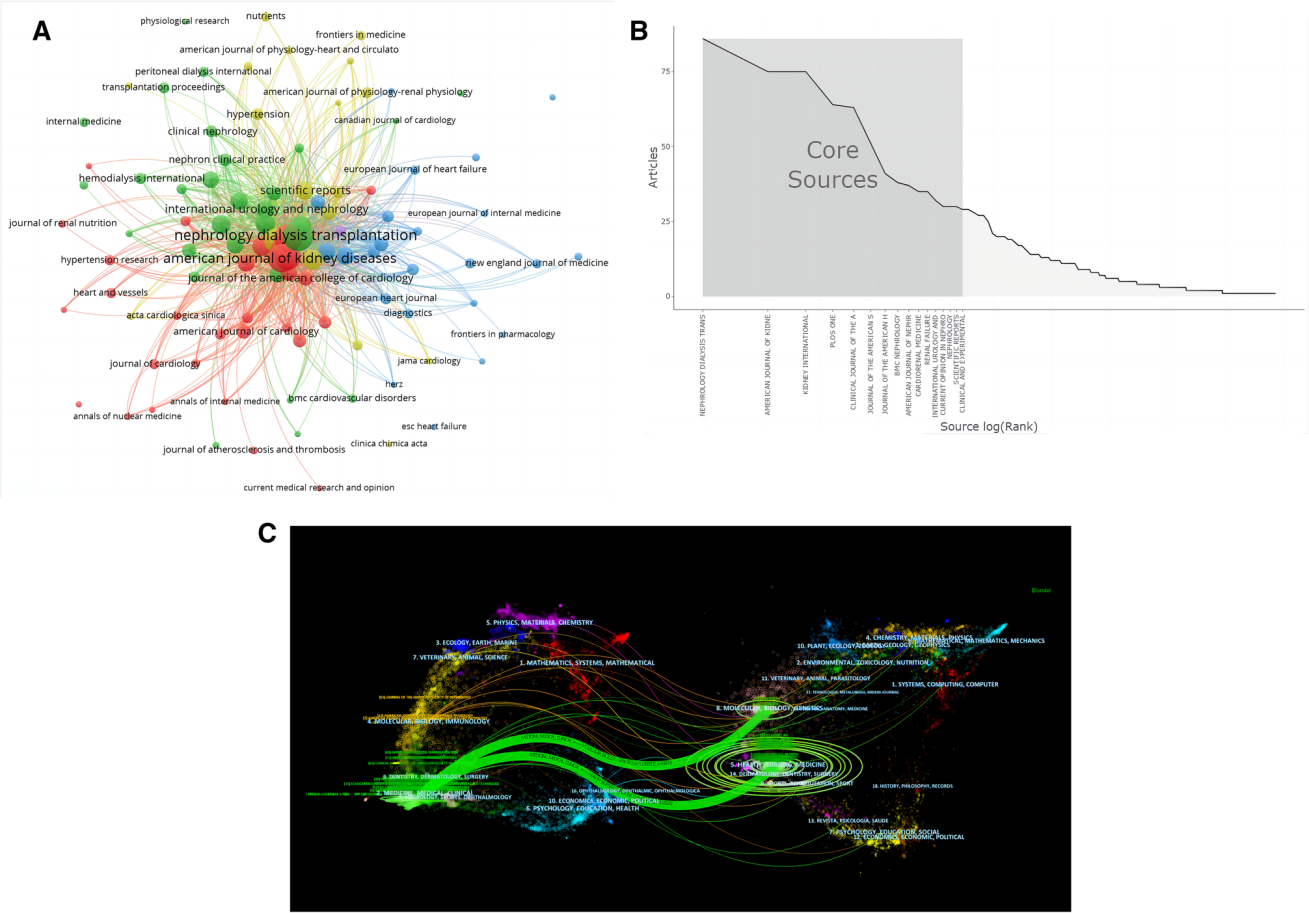
Journal analysis. **A** Journal distribution network. **B** Sources of core journals based on Bradford's law. **C** The dual-map overlay of the publications. (The left side is the citing journal, the right side is the cited journal, and the line path represents the citation relationship.)

A total of 451 journals were involved (Fig. 5A). According to Bradford's law, we defined the core area of the journal. They were mainly in the field of nephrology rather than cardiology (Fig. 5B). The journal with the most publications was *Nephrology Dialysis Transplantation* (Table 3). Journal dual-map coverage showed four major citation pathways in this field (Fig. 5C). The citing publications were concentrated in medicine, clinical and molecular fields. Other than that, the cited publications also focused on nursing and rehabilitation. The cited references involved 859 journals. Nine of the top ten most cited journals belonged to Q1 (Table 3). But it was worth noting that only half of the ten highest-circulation journals appeared in highly cited journals.

**Table 3:** Top 10 journals with the most publications and the most cited in the field of CRS-4

No.	Journal	Counts	IF/JCR	Cited journal	Counts	IF/JCR
1	NEPHROL DIAL TRANSPL	86	7.186/Q1	KIDNEY INT	1863	18.998/Q1
2	AM J KIDNEY DIS	75	11.072/Q1	CIRCULATION	1823	39.918/Q1
3	KIDNEY INT	75	18.998/Q1	J AM SOC NEPHROL	1810	14.978/Q1
4	PLOS ONE	64	3.752/Q2	AM J KIDNEY DIS	1743	11.072/Q1
5	CLIN J AM SOC NEPHRO	63	10.614/Q1	NEPHROL DIAL TRANSPL	1584	7.186/Q1
6	J AM SOC NEPHROL	51	14.978/Q1	NEW ENGL J MED	1555	176.079/Q1
7	J AM HEART ASSOC	41	6.106/Q2	J AM COLL CARDIOL	1543	27.203/Q1
8	BMC NEPHROL	38	2.583/Q3	CLIN J AM SOC NEPHRO	1088	10.614/Q1
9	AM J NEPHROL	37	4.605/Q1	LANCET	1032	202.731/Q1
10	CARDIORENAL MED	35	4.360/Q2	AM J CARDIOL	1012	3.133/Q3

### Keyword analysis

**Fig. 6:**
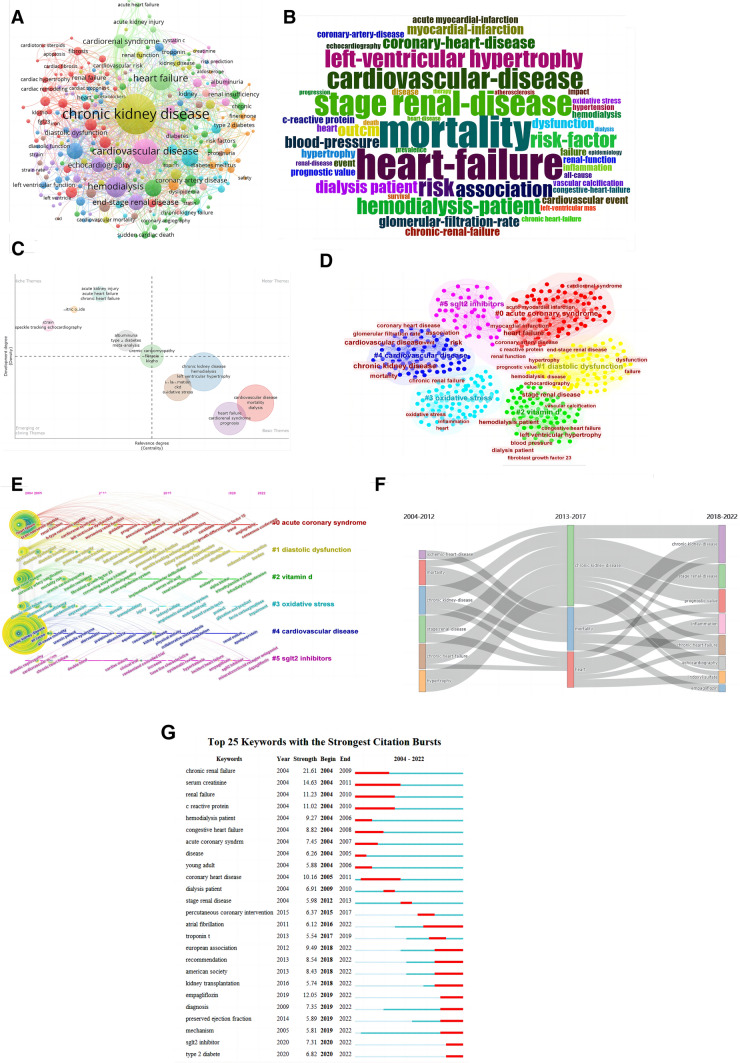
Keyword analysis. **A** Keyword network. **B** WordCloud. **C** Thematic Map. **D** Keyword clusters network. **E** Timeline plot of clustering based on keyword. **F** Thematic evolution. **G** Top 25 burst keywords

After combining keywords with the same meaning but different expressions (e.g. “chronic kidney disease” and “ckd”), we finally obtained 597 keywords (Fig. 6A, B). The most frequent keywords included “chronic kidney disease”, “cardiovascular disease”, and “heart failure”, which was consistent with the theme of our research. We also noticed that “mortality”, “stage renal disease” and “hemodialysis” were also the frequent-used keywords, suggesting that CRS-4 was closely related to the end-stage renal diseases and death in CKD patients.

In addition, we found that the keywords in this field were mainly distributed in the second and fourth quadrants of the theme map (Fig. 6C). This reflected to a certain extent that the foundation of CRS-4 development was not solid and the research focus had shifted. It was remarkable that uremic cardiomyopathy and klotho spanned four quadrants and might be the emerging focus of attention in the future.

On this basis, six keyword co-occurrence clusters were obtained (Fig. 6D). Most clusters were associated with cardiovascular events, such as #1 diastolic dysfunction and #4 cardiovascular disease, which was consistent with our research topic. Clusters #2 vitamin d and #5 sglt2 inhibitors suggested therapeutic research hotspots for CRS-4. Cluster #3 oxidative stress indicated the key pathophysiological characteristics of CRS-4.

We observed the timeline view meanwhile (Fig. 6E). Among early studies, the most extensive topic was cluster #4 cardiovascular disease. Cluster #5 sglt2 inhibitors was emerging research hotspots in recent years, which was consistent with the clustering content of co-cited references (Fig. 4C). Of note, scholars' attention to the prognostic value had shown a rapid growth trend in the past five years (Fig. 6F).

Burst vocabulary refers to the keywords that are widely concerned over a period of time, showing the evolution of hot topics (Fig. 6G). We found that the focus of disease research had gradually shifted from “chronic renal failure” “atorvastatin” to “type 2 diabetes” “sglt2 inhibitors”. This reflected that the interaction between diabetes mellitus (DM) and CRS-4 had attracted some attention in recent years. In addition, we noticed that “preserved ejection fraction” had also become the hot topic discussed recently.

## Discussion

In this study, we revealed the related research structure and progress in the field of CRS-4. The results showed that the annual publications on CRS-4 basically increased (Fig. 1). Cardiac damage in CKD patients began to be concerned in 1836 [4]. In 2004, GO AS et al. [11] demonstrated the close association between reduced glomerular filtration rate and cardiovascular events. Subsequently, in the consensus conference held by the ADQI in 2008, Ronco et al. [18] proposed the definition and classification of CRS-4, and provided potential management strategies. To the best of our knowledge, our study was the first to systematically analyze the hotspots and new trends in CRS-4 via bibliometrics. The USA was the largest contributor (Fig. 2B), with 9 of the top 10 production institutions from the USA (Table 1). Although China started late (Fig. 2E), it had the advantage of a large population base of CKD and contributed a lot to the development of CRS-4 research. The close cooperation between North America and Europe led and promoted the research and progress of CRS-4 (Fig. 2C). Our result was consistent with the study from Lv et al. [21] which analyzed the knowledge structure of all subtypes of CRS. RONCO C, GO AS, BANSAL N, CHEN J, and AGARWAL R were noteworthy authors (Fig. 3A, C), and Johns Hopkins University was a noteworthy publisher (Fig. 3B). Most of the co-cited references were reviews or guidelines from kidney/heart journals or top journals (Fig. 4A, B, and Table 2), of those, the journals concerning nephrology had a higher academic influence instead of cardiology (Fig. 5B).

Oxidative stress and inflammation are still the hot spots of CRS-4 research (Fig. 6C, D). Increased production of reactive oxygen species, reduced clearance of pro-oxidants and impaired antioxidant defenses can lead to oxidative stress and inflammatory response, which contribute a lot to the development of CKD [22, 23]. Of note, activation of the renin–angiotensin–aldosterone system and sympathetic nervous system can also amplify oxidative stress and inflammation in CKD patients [24]. These form a vicious circle, thereby exacerbating the endothelial injury, arteriosclerosis, and fibrosis, leading to various clinical complications such as CRS-4 and cerebral vascular diseases. Soluble growth stimulating gene 2 (sST2) and galectin-3 (Gal-3) are related indicators of fibrosis and inflammation, which have been intensively studied in recent years, revealing a strong diagnostic and prognostic evaluation for CRS-4 [25].

Interestingly, uremic toxins have attracted wide attention over the past 20 years (Fig. 6A, B), especially its impact on CKD, CVD, and CRS-4. Retention of uremic toxins was caused by decreased kidney function. Accumulating studies have demonstrated that uremic toxins can cause vascular inflammation and endothelial dysfunction as pro-oxidative and pro-inflammatory mediators, thus causing cardiovascular damage [26]. Our previous study [27] also found that serum level of trimethylamine N-oxide (TMAO), a uremic toxin derived mainly from dietary choline, was positively associated with serum levels of IL-1β and TNF-α in a CRS-4 rat model.

Fibroblast growth factor 23 (FGF23) and klotho are emerging trends in recent years (Figs. 4C, 6C). As a phosphate hormone secreted primarily by osteocyte, FGF23 controls metabolism of phosphate and vitamin D and maintains its homeostasis through klotho mediation [24]. In the early stage of CKD, FGF23 can balance the increase of phosphate level, thereby reducing phosphate-induced vascular calcification. In the end stage, phosphate overload and vitamin D deficiency down-regulate klotho expression in the kidney, which in turn decreases FGF23 affinity to FGF receptors and results in FGF23 resistance. The complex environment of hyperphosphatemia, FGF23 excess, and vitamin D deficiency accelerates the development of hypertension, vascular calcification, and left ventricular hypertrophy [28].

Sodium glucose cotransporter 2 (SGLT2) inhibitors have become an emerging hotspot recently (Fig. 4C, 6D, E and G), which reduce glucose reabsorption by inhibiting the SGLT2 channel located in the proximal tubule of the kidney, thereby exerting a hypoglycemic effect [29]. It has attracted wide attention due to their excellent cardiorenal protection independent of lowering glucose. In the latest years, numerous studies have shown that SGLT2 inhibitors may attenuate cardiac injury by correcting oxidative stress and inflammation, regulating mitochondrial function, and balancing autophagy [30].

It is worth noting that more and more studies are shifting focus on the prevention and prognosis evaluation of cardiovascular events in CKD patients. Classic cardiac biomarkers, like troponin [31] and natriuretic peptide [32], are susceptible to renal dysfunction with limited use in CKD population. Therefore, exploring new biomarkers to identify high-risk CKD patients prone to CRS-4 becomes the hotspot, such as asymmetric dimethylarginine (ADMA) [33], nonoxidized parathyroid hormone (PTH) [34], sST2 and Gal-3 [25]. In addition, some noninvasive imaging plays an essential role in assessing heart and vascular abnormalities, for example multi-modality cardiac imaging [35], and cardiac magnetic resonance [36]. These techniques can assist in identifying early changes in myocardial tissue characterization [37, 38], which can be applied as indicators for early diagnosis and risk stratification of CRS-4.

Surprisingly, we noticed that in burst vocabulary (Fig. 6G), although “acute coronary syndrome” strength ranked relatively high, it was only concerned for 3 years. This may be related to the update of the CRS-4 definition. Before 2008, the diagnostic criteria of CRS were ambiguous. In 2008, the ADQI divided CRS into 5 subtypes, of which CRS-4 referred to CHD, HF, and ACS secondary to CKD [18]. However, after retrieving relevant literature, we found that after 2008, scholars paid more attention to HF instead of ACS in the CKD population. Unsurprisingly, the American Heart Association updating the classification of CRS-4 in 2019 [39], which showed that the clinical examples of CRS-4 included left ventricular hypertrophy and HF from CKD-associated cardiomyopathy, and did not mention acute cardiovascular event.

## Limitations

Some limitations should be pointed out in our study. First, studies that didn’t clearly distinguish different types of CRS were not included in this study, which may have a certain impact on the results. Second, studies focusing on signaling pathways or drugs with cardio-renal effects were not included if they did not address kidney-to-heart crosstalk. Finally, as we all know, CKD often accompanies with DM, hypertension, obesity, or other metabolic diseases. It is difficult to determine whether the primary event of cardiovascular events in those patients was CKD, DM, or others.

## Conclusions

Our study reveals hot spots and new trends in research on CRS-4, which can provide scholars with key information on determining directions for future research. A total of 735 authors from 543 institutions in 94 countries/regions participated in the publication of CRS-4 papers, which were mostly from North America and Europe. Most of the co-cited references were reviews or guidelines from kidney/heart specialist journals or top journals. The journals concerning nephrology had a high academic influence in this field. Oxidative stress and inflammation remained hot topics in CRS-4 research, as well as uremic toxins. Emerging trends in recent years included FGF23 and klotho. The latest frontier hot spot was SGLT2 inhibitors. Future research progress may focus on the prevention and prognosis assessment of CRS-4.

## Data Availability

The data generated in this study has been included in the article, and further inquiries can be directed to the corresponding author or the first author.
